# Introduction and reproducibility of an updated practical grading system for lumbar foraminal stenosis based on high-resolution MR imaging

**DOI:** 10.1038/s41598-021-91462-2

**Published:** 2021-06-07

**Authors:** Elisabeth Sartoretti, Michael Wyss, Alex Alfieri, Christoph A. Binkert, Cyril Erne, Sabine Sartoretti-Schefer, Thomas Sartoretti

**Affiliations:** 1 Institute of Radiology, Kantonsspital Winterthur, Brauerstrasse 15, 8401 Winterthur, Switzerland; 2 Faculty of Medicine, University of Zurich, Zurich, Switzerland; 3 Philips Healthsystems, Zurich, Switzerland; 4 Institute of Neurosurgery, Kantonsspital Winterthur, Winterthur, Switzerland; 5 Department of Radiology and Nuclear Medicine, Maastricht University Medical Center, Maastricht University, Maastricht, The Netherlands

**Keywords:** Anatomy, Rheumatology

## Abstract

In this paper we sought to develop and assess the reproducibility of an updated 6-point grading system for lumbar foraminal stenosis based on the widely used Lee classification that more accurately describes lumbar foraminal stenosis as seen on high-resolution MRI. Grade A indicates absence of foraminal stenosis. Grades B, C, D and E indicate presence of foraminal stenosis with contact of the nerve root with surrounding anatomical structures (on one, two, three or four sides for B, C, D and E respectively) yet without morphological change of the nerve root. To each grade, a number code indicating the location of contact between the nerve root and surrounding anatomical structure(s) is appended. 1, 2, 3 and 4 indicate contact of the nerve root at superior, posterior, inferior and anterior position of the borders of the lumbar foramen. Grade F indicates presence of foraminal stenosis with morphological change of the nerve root. Three readers graded the lumbar foramina of 101 consecutive patients using high-resolution T2w (and T1w) MR images with a spatial resolution of beyond 0.5 mm^3^. Interreader agreement was excellent (Cohen’s Kappa = 0.866–1). Importantly, 30.6%/31.6%/32.2% (reader 1/reader 2/ reader 3) of foramina were assigned grades that did not appear in the original Lee grading system (grades B and D). The readers found no foramen that could not be described accurately with the updated grading system. Thus, an updated 6-point grading system for lumbar foraminal stenosis is reproducible and comprehensively describes lumbar foraminal stenosis as seen on high-resolution MRI.

## Introduction

Lumbar foraminal stenosis is a frequent condition that may cause back pain and radiculopathy due to nerve root compression and irritation. In a healthy person the nerve root passing through the spacious foramen is surrounded by perineural fat^1–3^ which protects the nerve root from compression of the adjacent osseous, ligamentous and discal structures. Foraminal stenosis reduces the space surrounding the nerve root and thus may cause nerve root contact and even nerve root compression with subsequent clinical symptomatology due to nerve root irritation.

MRI is considered the imaging modality of choice to visualize and evaluate lumbar foraminal stenosis^3,4^.

The degree of lumbar foraminal stenosis on MRI is best described with practical, qualitative grading systems as quantitative systems often suffer from lack of reproducibility^5^. Wildermuth et al.^1^ and Kunogi and Hasue^6^ were among the first to describe practical grading systems, but their approach exhibited certain limitations. More recently Lee et al. have developed a new grading system that overcomes previous limitations by combining key elements of prior grading systems. This grading system has gained widespread clinical use as it is simple, comprehensive and as it ensures a high degree of reproducibility. Until recently, we have also utilized this grading system at our institution to describe lumbar foraminal stenosis in our radiological reports.

With recent advances in MRI technology^7^, spine MRI protocols can be considerably improved either in spatial resolution or in reduction of scan time. Specifically, at our institution, sagittal 2-dimensional (2D) sequences with 3–4 mm slice thickness could be replaced by high-resolution 3-dimensional (3D) sequences that provide images and multiplanar reconstructions in submillimeter resolution.

The widely used grading system of Lee bases on the anatomical situation of the lumbar spinal nerve root within the intervertebral foramina as observed on sagittal 2D images^3^. With a slice thickness of 3–4 mm, the foramen is usually depicted on 2–3 sagittal images. With new high-resolution 3D MRI techniques, 9–21 sagittal slices depict the space of the intervertebral foramen^7^.

Thus, using high-resolution imaging which also allows for a reduction in partial volume effects as compared to the original thick 2D sagittal images, a much more complex relationship between the nerve root and the surrounding structures within the foramen can be identified. To this extent, an updated classification scheme has been developed at our institution in an effort to more accurately describe lumbar foraminal stenosis as seen on high-resolution imaging. This updated scheme builds on the original Lee grading system but is more detailed and accurately describes even the smallest anatomical changes in the lumbar foramen that would not have been visible on the original 2D sequences.

In this study, we sought to assess the reproducibility of this updated grading system, to compare it with the original Lee grading system and to discuss its potential clinical relevance.

## Materials and methods

### Development of an updated MRI grading system for lumbar foraminal stenosis

The updated grading system was developed in accordance with a senior neurosurgeon, two senior radiologists and two trainees who are all involved in the assessment of lumbar spine MRI examinations. The grading system was developed primarily based on a 3D sagittal high-resolution T2-weighted (T2w) sequence and secondarily on a 2D T1-weighted (T1w) sequence^3^.

**Figure 1:**
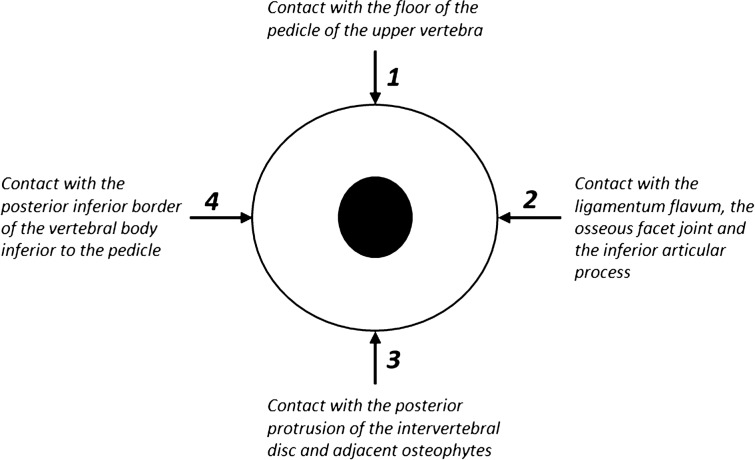
Illustration of the lumbar foramen and explanation of positional information.

The original Lee classification considers contact between nerve root and the surrounding structures in anterior and posterior transverse and in superior and inferior vertical direction due to disc space narrowing, discoosteophytic protrusions, thickened ligamentum flavum and facet arthropathy followed by nerve root collapse or morphologic nerve root change^3^. Thus, contact of the nerve root with the surroundings is possible at 4 different nerve root positions i.e. superior, posterior, inferior and anterior border. Superior contact (Fig. 1, arrow 1) is between nerve root and floor of the pedicle of the upper vertebra of the corresponding segment. Posterior contact (Fig. 1, arrow 2) is given by the ligamentum flavum and the osseous facet joint and the inferior articular process. Inferior contact (Fig. 1, arrow 3) is due to posterior protrusion of the intervertebral disc and adjacent osteophytes. Anterior contact (Fig. 1, arrow 4) is given by the posterior inferior border of the vertebral body inferior to the pedicle.

For our upgraded grading system, the original Lee classification was extended by two categories:

**Grade A:** Absence of foraminal stenosis (corresponding to Lee Grade 0).

**Grade B:** Very mild foraminal stenosis with perineural fat obliteration surrounding the nerve root in only one direction. It involves contact with a single position of the nerve root. There is no visible morphological change of the nerve root.

**Grade C:** Mild foraminal stenosis with perineural fat obliteration surrounding the nerve root in two directions. It involves contact with two positions of the nerve root. There is no visible morphological change of the nerve root (corresponding partially to Lee Grade 1).

**Grade D:** Moderate foraminal stenosis with perineural fat obliteration surrounding the nerve root in three directions. It involves contact with three positions of the nerve root. There is no visible morphological change of the nerve root.

**Grade E:** Severe foraminal stenosis with perineural fat obliteration surrounding the nerve root in four directions. It involves contact with four positions of the nerve root. There is no visible morphological change of the nerve root (corresponding to Lee Grade 2).

**Grade F:** Very severe foraminal stenosis with nerve root collapse or morphological change (corresponding partially to Lee Grade 3).

**Figure 2:**
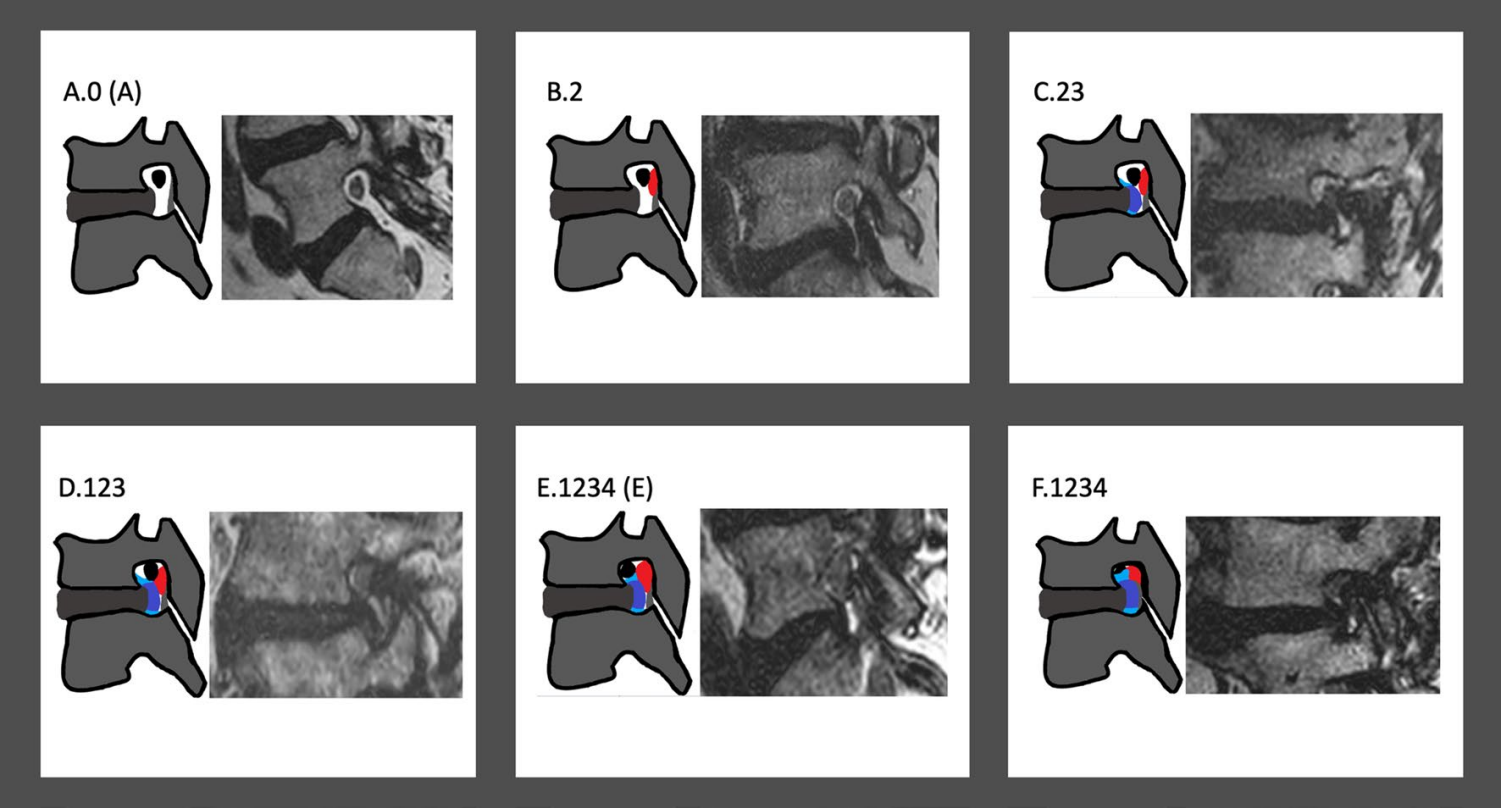
Schematic illustrations and imaging examples of relevant cases described in the updated grading system. The color red signifies the ligamentum flavum; the color dark blue signifies the posterior disc protrusion and the color light blue signifies the adjacent osteophytes.

Additionally, for each of the 6 categories, positional information of the nerve root contact is graded and appended to the letter (indicating the category) after a dot. This positional information is indicated with 4 numbers (1,2,3,4) and refers to the point of contact of the nerve root with surrounding structures within the foramen (except for fat) in clockwise direction. Position 1 means superior, position 2 means posterior, position 3 means inferior and position 4 means anterior contact of the nerve root with the surrounding structures (Fig. 1). Exemplary visual illustrations and imaging examples of all grades of stenosis described in this updated grading system are provided in Fig. 2.

**Figure 3:**
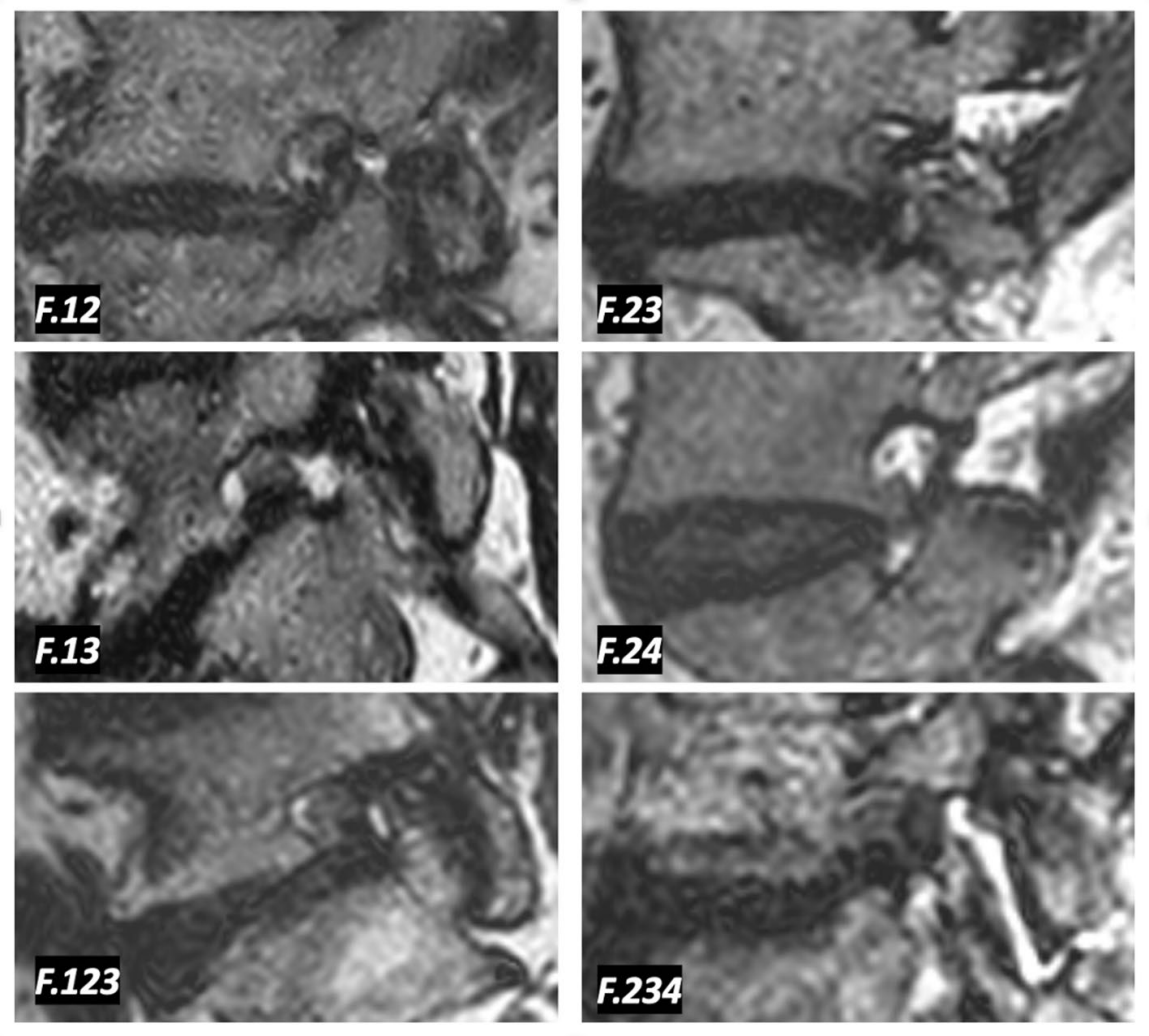
Representative imaging examples of grade F lumbar foraminal stenosis.

In case of Grade F lumbar foraminal stenosis, the number code appended after the letter does not only transmit positional information, but potentially also provides information on the severity of the morphological change of nerve root collapse. Specifically, a stenosis graded as F.1234 indicates that the collapsed nerve root is compressed from all 4 directions thus indicating the severest form of stenosis. Representative imaging examples of grade F stenosis are provided in Fig. 3.

Lastly, it should be noted that with this updated grading system, every possible combination of contact of the nerve root (and thus every possible situation) within the foramen can be described. Furthermore, as outlined above, our updated system is merely an extension of the original Lee classification and thus encompasses all the original grades described by Lee et al. This ensures, that our updated grading system can be applied in every situation, irrespective of MRI sequence parameters and spatial resolution of sequences.

### MR imaging

All patients underwent routine lumbar MRI examinations at one of three 1.5 T scanners (Achieva and Ingenia, Philips) at two centers. Patients were examined in supine position with slightly flexed knees.

Sequence parameters were as follows:

(a) 3D T2-weighted (w) Turbo Spin Echo (TSE) sequence: acquisition sagittal, DRIVE pulse yes, repetition time (TR) 1300 ms, echo time (TE) 95 ms, flip angle 90°, field of view (FOV) 200 × 300 × 90 mm^3^, acquired voxel size 0.8 × 0.8 × 1.0 mm^3^, reconstructed voxel size 0.4 × 0.4 × 0.5 mm^3^, number of slices 180, acceleration: Compressed SENSE factor 7.0, number of signal averages (NSA) 1.0, acquisition time 04 min 46 s. The sequence was generally acquired in sagittal plane of section parallel to the spinal column. In patients with scoliosis, however, a secondary curved sagittal reconstruction parallel to the scoliotic lumbar vertebrae was obtained.

(b) 2D T1w TSE sequence: acquisition sagittal, TR 502 ms, TE 8 ms, flip angle 90°, FOV 160 × 270 × 65 mm^3^, acquired voxel size 0.75 × 0.95 × 4.0 mm^3^, reconstructed voxel size: 0.63 × 0.63 × 4.0 mm^3^, number of slices 15, NSA 2.0, acquisition time 03 min 04 s. The sequence was acquired in sagittal plane of section parallel to the spinal column.

### Study subjects

This retrospective study was performed in accordance with the Declaration of Helsinki. The study was approved by the institutional review board (Cantonal Ethics Committee Zürich, Switzerland) and all methods were carried out in accordance with relevant guidelines and regulations. All study subjects provided general written informed consent. We analyzed all consecutive lumbar spine MRI examinations that had been performed at two radiological institutions between October and December 2020. In accordance with Lee et al.^3^ we applied the following exclusion criteria: (1) Patients below the age of 60 years. (2) Patients presenting with infections, tumors or fractures on MRI. (3) Patients who had undergone previous lumbar spine operations. After applying the exclusion criteria, we identified 101 eligible patients (average age: 73 years; age range: 60–91 years; 54 men; 47 women), which were subsequently included in the study. 94 patients were examined on one of two MR scanners (Achieva (n = 56), Ingenia (n = 38), both Philips) at institution 1 (Cantonal Hospital Winterthur, Switzerland) and 7 patients were examined on one MR scanner (Ingenia (n = 7), Philips) at institution 2 (WIN4, Switzerland).

### Image analysis

Three readers (board-certified neuroradiologist with 30 years of experience (reader 2) and two trainees each with 3 years of experience (readers 1 and 3)) analyzed all images independently in a blinded and randomized manner. The readers assessed and graded the presence of foraminal stenosis according to the updated grading system on sagittal T2w images and verified the findings on the T1w images in cases of suspicion of intraforaminal perineural cysts. Readers were allowed to freely scroll through all the images depicting the lumbar foramen to achieve a comprehensive 3-dimensional view of the nerve root within the foramen. This subsequently served as the basis for assigning the classification grades. In case a patient presented with foraminal disk extrusion with superior migration, the respective segment was excluded from image analysis^3^. The medial and lateral border of the intervertebral foramen corresponded to the medial and lateral border of the pedicle if sagittal images were correlated with transverse images^8^. Per patient 10 foramina ranging from L1-L2 to L5-S1 were evaluated (5 on each side). Lastly, readers were also asked to highlight cases where they felt that the updated grading system could not accurately classify or describe the degree of lumbar foraminal stenosis.

### Statistical analysis

Interreader agreement was quantified with Kappa statistics^3,9^. A kappa value of less than 0.20 was considered slight; 0.21–0.40, fair; 0.41–0.60, moderate; 0.61–0.80, substantial; and 0.81 or greater, nearly perfect agreement^3,10^. All analyses were performed in the R programming language (version 4.0.2) (R Core Team, 2020) using the package ”irr”.

## Results

A detailed overview of interreader agreement is provided in Table 1. In brief, the readers achieved a high degree of agreement with kappa values ranging from 0.866 to 1. In no case did the readers feel that the updated grading system could not accurately describe the degree of lumbar foraminal stenosis.

**Table 1:** Overview of interreader agreement.

Interreader agreement (Cohen's kappa)	L1-L2 (Left n = 100; Right n = 99)	L2-L3 (Left n = 101; Right n = 100)	L3-L4 (Left n = 96; Right n = 93)	L4-L5 (Left n = 91; Right n = 97)	L5-S1 (Left n = 93; Right n = 96)
Reader 2–Reader 3	Left: 0.91 Right: 0.915	Left: 0.933 Right: 0.94	Left: 0.963 Right: 0.975	Left: 0.938 Right: 0.905	Left: 0.976 Right: 0.887
Reader 1–Reader 2	Left: 0.964 Right: 0.935	Left: 0.96 Right: 0.97	Left: 1 Right: 0.937	Left: 0.938 Right: 0.906	Left: 0.988 Right: 0.955
Reader 1–Reader 3	Left: 0.946 Right: 0.933	Left: 0.946 Right: 0.97	Left: 0.963 Right: 0.912	Left: 0.975 Right: 0.906	Left: 0.965 Right: 0.886

Out of 966 foramina, Grade A was chosen in 34.1%, 32.7% and 33.5% of cases respectively (reader 1, reader 2, reader 3). Grade B was selected in 18.9%, 19.8% and 19.8% of cases. Grade C was determined in 25.2%, 25.7% and 24.3% of cases. Grade D was present in 11.7%, 11.8% and 12.4% of cases. Grade E was selected in 3.62%, 3.52 and 3.42% of cases. Finally, Grade F was chosen by all readers in 6.52% of cases.

As for the comparison with the original Lee grading system, 30.6%, 31.6% and 32.2% of cases did not correspond directly with grades that were present in the original Lee grading system (i.e. Grades B and D are not present in the original Lee system). A detailed overview of the data is presented in the supplementary material.

## Discussion

In this feasibility study we assessed the reproducibility of an updated practical grading system for lumbar foraminal stenosis based on high-resolution MRI.

We provide evidence, that this updated system exhibits a high degree of reproducibility and comprehensively enables the description of even the smallest anatomical changes in the lumbar foramen as observed on high-resolution MRI.

The 4-point grading system by Lee et al. considers both the degree of perineural fat obliteration as well as the nerve root morphology. This classification has been shown to enable a comprehensive description of lumbar foraminal stenosis with a high degree of interreader agreement (kappa values ranging from 0.8 to 1)^3^. Furthermore, this grading system has been shown to be of clinical value as clinical findings could partially be correlated with radiological grading scores^4^. Interestingly, a CT version of this system was described recently, which correlates well with the MRI version and also exhibits a high degree of reproducibility^11^.

In the original study by Lee et al. conventional and widely used^12–14^ 2D T2w and T1w TSE sequences with slice thicknesses of 4 mm and slice gaps of 0.4 mm were used^3^. Thus, the foramen is only depicted on one to three image slices which impacts the detectability of more subtle changes within the foraminal zone. In our study we used a modern, clinically available compressed sensing (CS) accelerated^15–17^ 3D T2w MRI sequence with reconstructed voxel sizes of beyond 0.5 mm^3^ at a scan time comparable to the original 2D sequence. On these high-resolution images the entire path of the nerve root within the lumbar foramen is visualized in much more detail thus depicting the nerve root within the foramen on about 19 to 21 sagittal images. Moreover, partial volume effects do not disturb image interpretation and careful scrolling through subsequent images allows a highly detailed morphological analysis of the relationship between the nerve root and its foraminal surroundings. Out of 966 lumbar foramina, approximately 30% of cases were graded with categories that did not appear in the original Lee classification. This corroborates the need for an updated classification system suitable for high resolution thin slice images in the submillimeter range thus enabling the radiologist to fulfil his duty to describe and classify radiological images as accurately as possible. Additionally, when considering the prevalence of back pain and clinical symptoms presumably originating from radiculopathy^6,18,19^, an updated grading system describing even subtle changes with the lumbar foramen may be highly desirable. This is further supported by the fact that there is still urgent need for improving the correlation of clinical symptoms and imaging findings^4,20^.

Importantly, we would like to highlight that this updated grading system fully leverages the potential of modern MRI techniques with spatial resolutions riveling those achieved in CT imaging yet at excellent soft tissue contrast^11^. While a CT version of the Lee grading system has been described recently^11^, a further study should investigate whether our updated grading system can also be translated to CT imaging.

In this regard it would also be interesting to clarify to what extent the upgraded grading system can be applied to conventional 2D images with a slice thickness of 3-4 mm. The original Lee system was developed and validated on conventional 2D images and our upgraded system incorporates all the original grades from the Lee system. Thus, per definition, our upgraded system can also be applied for conventional 2D images. However, it is unclear whether readers would assign the same grades from the upgraded system for the 2D and 3D images. Specifically, should the assignment of grades differ between conventional 2D and high-resolution 3D imaging, it would be interesting to see whether this has an influence on the further course of therapy. To test this, one would have to perform an intra-individual comparison, preferably in patients in whom both conventional 2D T2w and high-resolution 3D T2w images were acquired in the same MR examination. This should be addressed and analyzed in future studies.

On another note, we hypothesize that our updated grading system may also be of value for research applications as the positional information also provides insight into the pathophysiology of the condition. In other words, information can be gained on what anatomical structures are most frequently involved in lumbar foraminal stenosis, simply by deciphering the grading code.

Ultimately, however, it must be clarified whether the updated classification system really does bring benefits in terms of clinical correlation. We think that the detailed description of certain imaging findings probably does not provide clinical benefits. For example, we consider it unlikely that grade B changes can be clinically distinguished from grade A changes. However, the fine division could still be clinically relevant, namely in terms of prognostic implications. In other words, we hypothesize that grade D changes may be at a higher risk of developing into high-grade stenosis grade E and F than grade C changes. Thus, this updated grading system offers a certain flexibility due to the wealth of information. Future studies must define which information is clinically relevant. Either way, given the fact that the updated grading system encompasses all the original categories by Lee, we expect that the updated system will not perform worse than the original Lee system in terms of clinical correlation. However, as indicated above, this must be addressed in further studies.

Our study has certain limitations: As mentioned before, our grading system is based on static sagittal MR images without symptomatic correlation. Specifically, clinical symptoms may arise only with dynamic changes, such as lumbar extension, which cannot be provoked or detected in a closed MR system^3,11^. Furthermore we primarily used 3D T2w images for grading because this sequence could be acquired (nearly isotropic) in high resolution at a reasonable scan time with the possibility to get curved transverse and coronal reconstructions. We are aware that T1w images may be preferred at other institutions due to the high contrast between fat and the surrounding tissues. However, a high contrast between fat and surrounding tissues was also achieved in our 3D T2w sequence.

In summary, we present an updated grading system for lumbar foraminal stenosis based on high-resolution MRI that is reproducible and comprehensively describes even the smallest anatomical changes in the lumbar foramen. Further studies must assess whether the wealth of information provided in the updated grading system may be of clinical utility.

## Supplementary Information

Supplementary Information.

## Data Availability

Data can be made available upon reasonable request to the corresponding author.

## Abbreviations

MRI: Magnetic Resonance Imaging
2D: 2-Dimensional
3D: 3-Dimensional
