# Author Correction: Introduction and reproducibility of an updated practical grading system for lumbar foraminal stenosis based on high-resolution MR imaging

**DOI:** 10.1038/s41598-021-98469-9

**Published:** 2021-09-15

**Authors:** Elisabeth Sartoretti, Michael Wyss, Alex Alfieri, Christoph A. Binkert, Cyril Erne, Sabine Sartoretti‑Schefer, Thomas Sartoretti

**Affiliations:** 1 Institute of Radiology, Kantonsspital Winterthur, Brauerstrasse 15, 8401 Winterthur, Switzerland; 2 Faculty of Medicine, University of Zurich, Zurich, Switzerland; 3 Philips Healthsystems, Zurich, Switzerland; 4 Institute of Neurosurgery, Kantonsspital Winterthur, Winterthur, Switzerland; 5 Department of Radiology and Nuclear Medicine, Maastricht University Medical Center, Maastricht University, Maastricht, The Netherlands

Correction to: *Scientific Reports* 10.1038/s41598-021-91462-2, published online 07 June 2021

The original version of this Article contained errors.

In the Materials and methods section, under the subheading ‘Development of an updated MRI grading system for lumbar foraminal stenosis’, the numbers given for the Lee Grade classification were incorrect.

As a result,

**“Grade A:** Absence of foraminal stenosis (corresponding to Lee Grade 1).”

now reads:

**“Grade A:** Absence of foraminal stenosis (corresponding to Lee Grade 0).”

And,

**“Grade C:** Mild foraminal stenosis with perineural fat obliteration surrounding the nerve root in two directions. It involves contact with two positions of the nerve root. There is no visible morphological change of the nerve root (corresponding partially to Lee Grade 2).”

now reads:

**“Grade C:** Mild foraminal stenosis with perineural fat obliteration surrounding the nerve root in two directions. It involves contact with two positions of the nerve root. There is no visible morphological change of the nerve root (corresponding partially to Lee Grade 1).”

And,

**“Grade E:** Severe foraminal stenosis with perineural fat obliteration surrounding the nerve root in four directions. It involves contact with four positions of the nerve root. There is no visible morphological change of the nerve root (corresponding to Lee Grade 3).”

now reads:

**“Grade E:** Severe foraminal stenosis with perineural fat obliteration surrounding the nerve root in four directions. It involves contact with four positions of the nerve root. There is no visible morphological change of the nerve root (corresponding to Lee Grade 2).”

And,

**“Grade F:** Very severe foraminal stenosis with nerve root collapse or morphological change (corresponding partially to Lee Grade 4).”

now reads:

**“Grade F:** Very severe foraminal stenosis with nerve root collapse or morphological change (corresponding partially to Lee Grade 3).”

Furthermore, Figure 2 contained errors in the top row where “B.2” was incorrectly given as “B.1” and “C.23” was incorrectly given as “C.12”.

Additionally, the Figure legend was incomplete.

“Schematic illustrations and imaging examples of relevant cases described in the updated grading system.”

now reads:

“Schematic illustrations and imaging examples of relevant cases described in the updated grading system. The color red signifies the ligamentum flavum; the color dark blue signifies the posterior disc protrusion and the color light blue signifies the adjacent osteophytes.”

The original Figure 2 and accompanying legend appear below.

**Figure 2:**
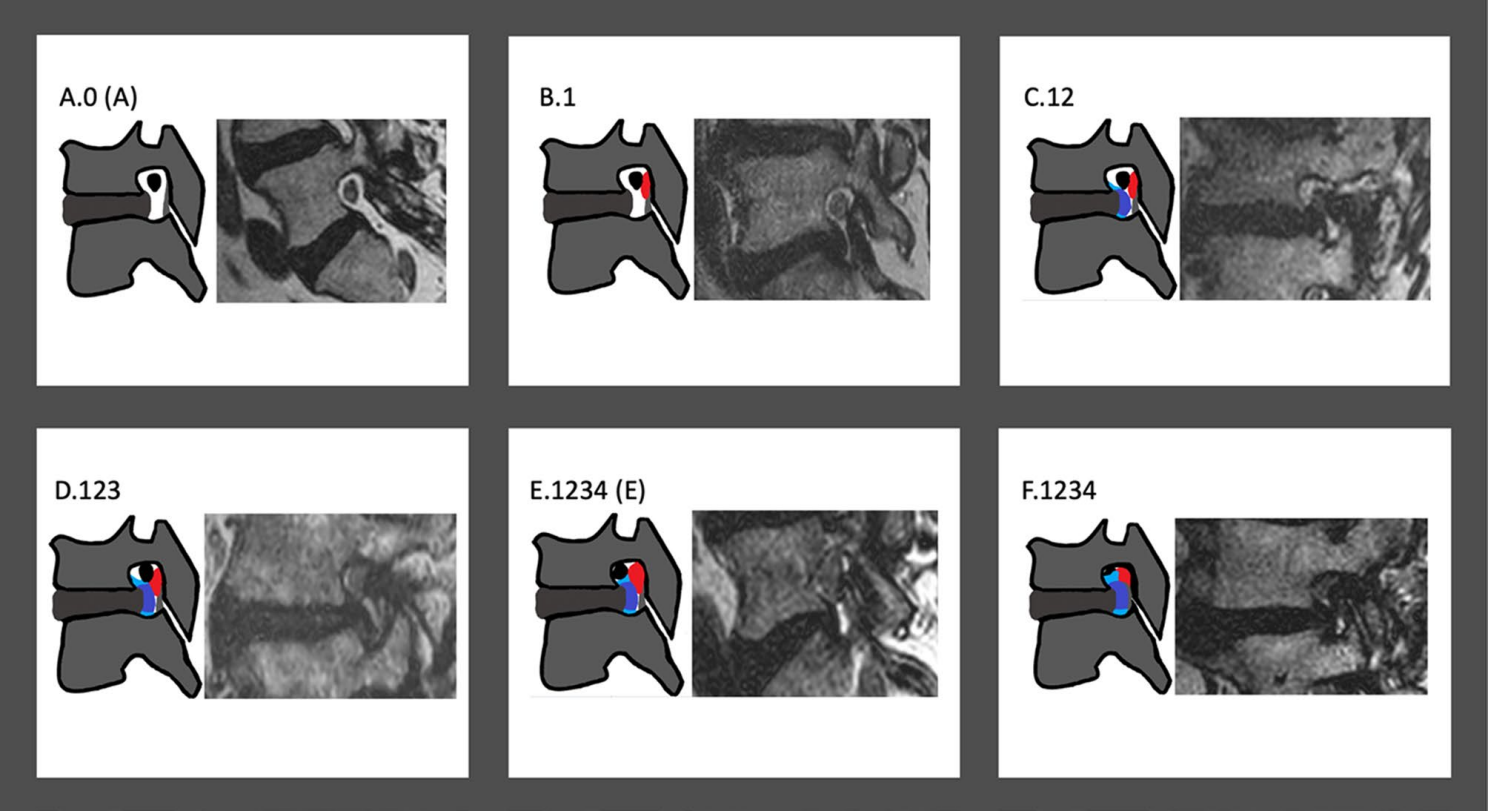
Schematic illustrations and imaging examples of relevant cases described in the updated grading system.

The original Article has been corrected.

