# Supplementary material for: Introduction and reproducibility of an updated practical grading system for lumbar foraminal stenosis based on high-resolution MR imaging
Source: Sci Rep. 2021 Jun 7;11:12000. doi: 10.1038/s41598-021-91462-2 (PMC8184791; doi:10.1038/s41598-021-91462-2)
Supplement: Supplementary file 1 — Supplementary Information. [file 41598_2021_91462_MOESM1_ESM.docx]

**Introduction and Reproducibility of an Updated Practical Grading System for Lumbar Foraminal Stenosis based on High-Resolution MR Imaging**

Elisabeth Sartoretti, Michael Wyss, Alex Alfieri, Christoph A. Binkert, Cyril Erne, Sabine Sartoretti-Schefer, Thomas Sartoretti

**Supplementary Material**

Detailed overview of the grading scores of each reader stratified by grading category, spinal segment and side.

| Reader 2 left side (n=481) | L1-L2 | L2-L3 | L3-L4 | L4-L5 | L5-S1 |
| --- | --- | --- | --- | --- | --- |
| A.0 | 64 | 43 | 21 | 12 | 12 |
| B.1 | 7 | 16 | 8 | 7 | 9 |
| B.2 | 13 | 10 | 6 | 3 | 1 |
| B.3 | 0 | 2 | 1 | 5 | 4 |
| B.4 | 0 | 0 | 1 | 0 | 2 |
| C.12 | 14 | 18 | 23 | 19 | 2 |
| C.13 | 0 | 2 | 5 | 8 | 12 |
| C.14 | 1 | 0 | 0 | 3 | 4 |
| C.23 | 0 | 2 | 8 | 3 | 3 |
| C.24 | 0 | 0 | 0 | 1 | 0 |
| C.34 | 0 | 0 | 0 | 0 | 3 |
| D.123 | 0 | 5 | 15 | 15 | 12 |
| D.124 | 0 | 0 | 2 | 0 | 1 |
| D.134 | 0 | 0 | 0 | 1 | 8 |
| D.234 | 0 | 1 | 0 | 0 | 0 |
| E.1234 | 0 | 0 | 1 | 5 | 8 |
| F.12 | 0 | 0 | 0 | 1 | 0 |
| F.123 | 0 | 0 | 0 | 2 | 1 |
| F.1234 | 1 | 2 | 4 | 1 | 7 |
| F.124 | 0 | 0 | 0 | 1 | 0 |
| F.13 | 0 | 0 | 0 | 0 | 3 |
| F.134 | 0 | 0 | 0 | 1 | 0 |
| F.2 | 0 | 0 | 0 | 0 | 0 |
| F.23 | 0 | 0 | 1 | 2 | 1 |
| F.24 | 0 | 0 | 0 | 1 | 0 |

| Reader 2 right side (n=485) | L1-L2 | L2-L3 | L3-L4 | L4-L5 | L5-S1 |
| --- | --- | --- | --- | --- | --- |
| A.0 | 69 | 53 | 23 | 6 | 13 |
| B.1 | 3 | 12 | 9 | 10 | 10 |
| B.2 | 13 | 8 | 8 | 6 | 2 |
| B.3 | 0 | 0 | 0 | 2 | 9 |
| B.4 | 1 | 0 | 0 | 0 | 3 |
| C.12 | 9 | 17 | 13 | 19 | 5 |
| C.13 | 0 | 1 | 1 | 5 | 13 |
| C.14 | 2 | 0 | 0 | 1 | 3 |
| C.23 | 0 | 1 | 9 | 8 | 6 |
| C.24 | 0 | 0 | 0 | 0 | 0 |
| C.34 | 0 | 0 | 0 | 1 | 3 |
| D.123 | 0 | 2 | 15 | 23 | 9 |
| D.124 | 0 | 1 | 0 | 1 | 0 |
| D.134 | 0 | 0 | 0 | 0 | 3 |
| D.234 | 0 | 0 | 0 | 0 | 0 |
| E.1234 | 1 | 2 | 6 | 6 | 5 |
| F.12 | 0 | 1 | 1 | 0 | 1 |
| F.123 | 0 | 0 | 1 | 1 | 1 |
| F.1234 | 0 | 1 | 3 | 5 | 3 |
| F.124 | 0 | 1 | 0 | 0 | 1 |
| F.13 | 0 | 0 | 0 | 1 | 4 |
| F.134 | 0 | 0 | 0 | 1 | 1 |
| F.2 | 0 | 0 | 2 | 0 | 0 |
| F.23 | 0 | 0 | 1 | 1 | 1 |
| F.24 | 1 | 0 | 1 | 0 | 0 |

| Reader 3 left side (n=481) | L1-L2 | L2-L3 | L3-L4 | L4-L5 | L5-S1 |
| --- | --- | --- | --- | --- | --- |
| A.0 | 63 | 46 | 21 | 14 | 12 |
| B.1 | 9 | 13 | 8 | 7 | 9 |
| B.2 | 14 | 11 | 7 | 2 | 1 |
| B.3 | 0 | 2 | 1 | 4 | 5 |
| B.4 | 0 | 0 | 1 | 0 | 2 |
| C.12 | 10 | 17 | 22 | 19 | 3 |
| C.13 | 1 | 2 | 5 | 8 | 11 |
| C.14 | 2 | 0 | 0 | 3 | 4 |
| C.23 | 0 | 1 | 8 | 2 | 3 |
| C.24 | 0 | 0 | 0 | 1 | 0 |
| C.34 | 0 | 0 | 0 | 0 | 3 |
| D.123 | 0 | 5 | 15 | 15 | 12 |
| D.124 | 0 | 1 | 2 | 0 | 1 |
| D.134 | 0 | 0 | 0 | 1 | 7 |
| D.234 | 0 | 1 | 0 | 0 | 0 |
| E.1234 | 0 | 0 | 1 | 6 | 8 |
| F.12 | 0 | 0 | 0 | 1 | 0 |
| F.123 | 0 | 0 | 0 | 1 | 1 |
| F.1234 | 1 | 2 | 4 | 1 | 7 |
| F.124 | 0 | 0 | 0 | 1 | 0 |
| F.13 | 0 | 0 | 0 | 1 | 3 |
| F.134 | 0 | 0 | 0 | 1 | 0 |
| F.2 | 0 | 0 | 0 | 0 | 0 |
| F.23 | 0 | 0 | 1 | 2 | 1 |
| F.24 | 0 | 0 | 0 | 1 | 0 |

| Reader 3 right side  (n=485) | L1-L2 | L2-L3 | L3-L4 | L4-L5 | L5-S1 |
| --- | --- | --- | --- | --- | --- |
| A.0 | 71 | 54 | 23 | 7 | 13 |
| B.1 | 4 | 11 | 8 | 12 | 11 |
| B.2 | 12 | 9 | 7 | 5 | 2 |
| B.3 | 0 | 0 | 0 | 2 | 9 |
| B.4 | 1 | 0 | 0 | 0 | 2 |
| C.12 | 8 | 16 | 15 | 15 | 6 |
| C.13 | 0 | 1 | 1 | 5 | 11 |
| C.14 | 1 | 0 | 0 | 1 | 3 |
| C.23 | 0 | 1 | 9 | 8 | 5 |
| C.24 | 0 | 0 | 0 | 1 | 0 |
| C.34 | 0 | 0 | 0 | 0 | 3 |
| D.123 | 0 | 3 | 15 | 25 | 11 |
| D.124 | 0 | 1 | 0 | 1 | 0 |
| D.134 | 0 | 0 | 0 | 1 | 2 |
| D.234 | 0 | 0 | 0 | 0 | 1 |
| E.1234 | 1 | 1 | 6 | 5 | 5 |
| F.12 | 0 | 1 | 1 | 0 | 1 |
| F.123 | 0 | 0 | 1 | 1 | 0 |
| F.1234 | 0 | 1 | 3 | 5 | 4 |
| F.124 | 0 | 1 | 0 | 0 | 1 |
| F.13 | 0 | 0 | 0 | 1 | 4 |
| F.134 | 0 | 0 | 0 | 1 | 1 |
| F.2 | 0 | 0 | 2 | 0 | 0 |
| F.23 | 0 | 0 | 1 | 1 | 1 |
| F.24 | 1 | 0 | 1 | 0 | 0 |

| Reader 1 left side (n=481) | L1-L2 | L2-L3 | L3-L4 | L4-L5 | L5-S1 |
| --- | --- | --- | --- | --- | --- |
| A.0 | 64 | 45 | 21 | 14 | 12 |
| B.1 | 8 | 14 | 8 | 7 | 10 |
| B.2 | 13 | 10 | 6 | 2 | 1 |
| B.3 | 0 | 2 | 1 | 4 | 4 |
| B.4 | 0 | 0 | 1 | 0 | 1 |
| C.12 | 12 | 18 | 23 | 20 | 2 |
| C.13 | 0 | 2 | 5 | 8 | 12 |
| C.14 | 2 | 0 | 0 | 3 | 4 |
| C.23 | 0 | 2 | 8 | 2 | 3 |
| C.24 | 0 | 0 | 0 | 1 | 0 |
| C.34 | 0 | 0 | 0 | 0 | 3 |
| D.123 | 0 | 5 | 15 | 15 | 12 |
| D.124 | 0 | 0 | 2 | 0 | 1 |
| D.134 | 0 | 0 | 0 | 1 | 8 |
| D.234 | 0 | 1 | 0 | 0 | 0 |
| E.1234 | 0 | 0 | 1 | 5 | 8 |
| F.12 | 0 | 0 | 0 | 1 | 0 |
| F.123 | 0 | 1 | 0 | 1 | 1 |
| F.1234 | 1 | 1 | 4 | 1 | 7 |
| F.124 | 0 | 0 | 0 | 1 | 0 |
| F.13 | 0 | 0 | 0 | 1 | 3 |
| F.134 | 0 | 0 | 0 | 1 | 0 |
| F.2 | 0 | 0 | 0 | 0 | 0 |
| F.23 | 0 | 0 | 1 | 2 | 1 |
| F.24 | 0 | 0 | 0 | 1 | 0 |

| Reader 1 right side  (n=485) | L1-L2 | L2-L3 | L3-L4 | L4-L5 | L5-S1 |
| --- | --- | --- | --- | --- | --- |
| A.0 | 72 | 54 | 25 | 7 | 15 |
| B.1 | 2 | 11 | 7 | 11 | 10 |
| B.2 | 12 | 8 | 8 | 6 | 2 |
| B.3 | 0 | 0 | 1 | 2 | 8 |
| B.4 | 0 | 0 | 0 | 0 | 3 |
| C.12 | 9 | 17 | 13 | 16 | 5 |
| C.13 | 0 | 1 | 1 | 6 | 12 |
| C.14 | 2 | 0 | 0 | 1 | 2 |
| C.23 | 0 | 1 | 8 | 9 | 6 |
| C.24 | 0 | 0 | 0 | 0 | 0 |
| C.34 | 0 | 0 | 0 | 1 | 3 |
| D.123 | 0 | 3 | 15 | 20 | 10 |
| D.124 | 0 | 1 | 0 | 1 | 0 |
| D.134 | 0 | 0 | 0 | 0 | 3 |
| D.234 | 0 | 0 | 0 | 0 | 0 |
| E.1234 | 1 | 1 | 6 | 8 | 5 |
| F.12 | 0 | 1 | 1 | 0 | 1 |
| F.123 | 0 | 0 | 1 | 1 | 1 |
| F.1234 | 0 | 1 | 3 | 5 | 3 |
| F.124 | 0 | 1 | 0 | 0 | 1 |
| F.13 | 0 | 0 | 0 | 1 | 4 |
| F.134 | 0 | 0 | 0 | 1 | 1 |
| F.2 | 0 | 0 | 2 | 0 | 0 |
| F.23 | 0 | 0 | 1 | 1 | 1 |
| F.24 | 1 | 0 | 1 | 0 | 0 |
